# Pnma5 is essential to the progression of meiosis in mouse oocytes through a chain of phosphorylation

**DOI:** 10.18632/oncotarget.18425

**Published:** 2017-06-09

**Authors:** Xiao-Lan Zhang, Peng Liu, Zhi-Xia Yang, Jing-Jing Zhao, Lei-Lei Gao, Bo Yuan, Li-Ya Shi, Chun-Xiang Zhou, Hai-Feng Qiao, Ya-Hong Liu, Xiao-Yan Ying, Jun-Qiang Zhang, Xiu-Feng Ling, Dong Zhang

**Affiliations:** ^1^ State Key Laboratory of Reproductive Medicine, Nanjing Medical University, Nanjing, Jiangsu, China; ^2^ Nanjing Maternity and Child Health Care Hospital, Nanjing, Jiangsu, China; ^3^ Department of Veterinary Drug Supervision, Taiyuan, Shanxi, China; ^4^ Wenxi Agriculture Committee, Yuncheng, Shanxi, China; ^5^ Department of Obstetrics and Gynecology, The Second Affiliated Hospital of Nantong University, Nantong, Jiangsu, China; ^6^ Department of Obstetrics and Gynecology, The Second Affiliated Hospital, Nanjing Medical University, Nanjing, Jiangsu, China

**Keywords:** Pnma5, meiosis, oocytes, fertilization, phosphorylation

## Abstract

PNMA (paraneoplastic antigen MA) family includes Pnma1–6. Although other members have been found to be involved in paraneoplastic neurological disorders, death receptor-dependent apoptosis, and tumorigenesis, Pnma5 was thought to be a female fertility factor, as indicated by one genome-wide study. But until now there have not been any further functional studies about Pnma5 in female meiosis. Our preliminary study indicated that Pnma5 might play important roles in meiosis. To further address this, Pnma5 was knocked down in in-vitro maturated (IVM) mouse oocytes, which are common models for mammalian female meiosis, by specific siRNA, and results showed that the loss of Pnma5 significantly delayed the progression of meiosis I and increased chromosome segregation errors during anaphase I. In in-vitro fertilization (IVF), Pnma5 knockdown caused significantly lower fertilization. To assess how it affects meiosis, Pnma5 knockdown was found to significantly decrease the stability of spindle microtubules and altered F-actin organization within actin cap regions, cause significantly abnormal mitochondria aggregation and lower ATP concentration. Next we have found that phosphorylation at Thr533 re-located Pnma5 strongly to spindles & cortex and was required for the phosphorylation of Akt and Gsk3β, while Src and Erk1/2 phosphorylation was required for the phosphorylation of Pnma5, indicating that phosphorylated Pnma5 is the active form and subsequently activates Akt and Gsk3β. Collectively this study suggests that Pnma5 is important for meiosis and is the pivot of Src→Erk1/2→Pnma5→Akt→Gsk3β pathway.

## INTRODUCTION

Risk factors associated with declines in female fertility include inheritance, specific diseases, psychology, and environment [1–3]. Eventually all these things disturb normal meiosis, which is key to stable genetic passage. Maternal factors (mRNAs and proteins) are synthesized and accumulated in the process of follicular development and have significant impacts on oocyte maturation and early embryonic growth. Normally, they are stored in cytoplasm and start to work as fully potential oocytes and resume meiosis upon the stimulation of luteinizing hormone [4–6]. Recently multiple transcriptome and proteome-wide studies have identified many novel female fertility factors that are expressed exclusively in ovaries and might have indispensable functions in oocyte meiosis and follicle maturation, though the functions of most of them remain unknown [7, 8].

The paraneoplastic Ma (PNMA) family contains of six members Pnma1, Pnma2, Pnma3, Pnma4 (Moap1), Pnma5, and Pnma6A with a common PNMA domain [9–12]. Pnma1 and Pnma3 are pro-apoptotic proteins in neurons and highly expressed in perinatal brain and adult testis. They have been identified to be involved in mouse neuronal cell death (depends on a BH3-like domain) but they promote cell growth in human pancreatic ductal adenocarcinoma (PDAC) cell lines and tissues. Recent studies showed that PI3K/AKT, MAPK/ERK pathway and members of the anti-apoptotic Bcl-2 family may participate in the pro-survival and anti-apoptotic impact of Pnma1 and Pnma4 in brains and hearts [13, 14]. However, Pnma2 was found to resist pro-apoptotic and chemo-sensitivity mediated by Pnma4 and Pnma1 through heterodimeric interaction [15, 16]. In all, the Pnmas are different from each other but somewhat overlapping in expression, distribution, and function.

However, Pnma5 appears even more different from other Pnmas than they are from each other. For example, Pnma 1–4 share moderate to high conservation in amino acid sequences but Pnma5 is less conserved [17–22]. Pnma5 have distinct localization pattern from other Pnmas in primate brain [23]. And Pnma5 was thought to be a female fertility factor in a genome-wide study [24], but until now there have not been any further functional studies about Pnma5 in female meiosis. Preliminary examination showed that Pnma5 is the most abundant among Pnma family members in oocytes and much more prominent than in granulosa cells, indicating that its function might be related to oocyte meiosis. Meanwhile, mitochondria is the apparatus that provide ATP to drive all cellular processes including meiosis, and multiple studies have shown that mitochondria dysfunction is one of the key factors conducing to abnormal meiosis [25–27]. So we also verified that Pnma5 is required for mitochondria dynamics and function. The purpose of this study was trying to address whether and how it affects meiosis.

## RESULTS

### Pnma5 is an oocyte-predominant protein found in the ovary

**Figure 1:**
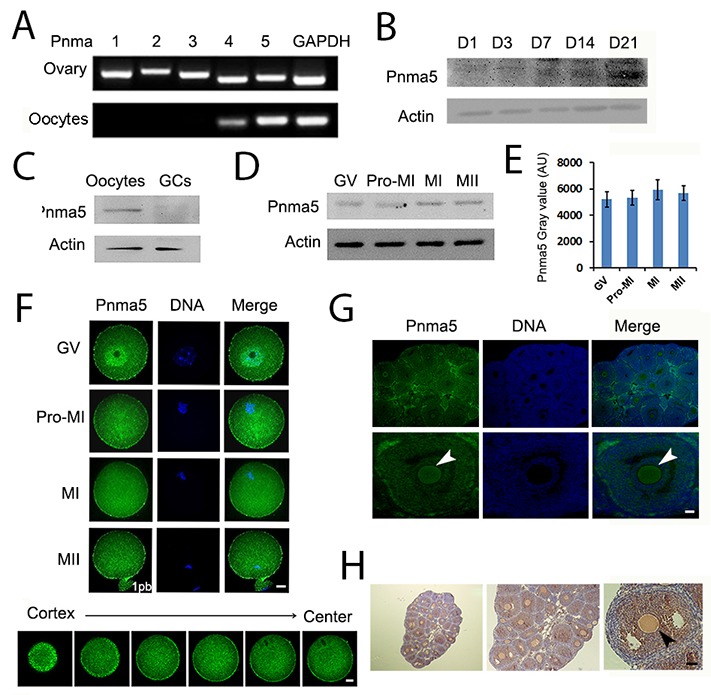
Pnma5 is an oocyte-predominant protein found in the ovary **(A)** RT-PCR showed that the mRNA level of PNMA 1∼5 in ovaries was close to each other, while in oocytes, PNMA5 has the strongest expression among them. **(B)** Pnma5 immunoblots of ovaries at different post-natal days (PND) showed that the protein levels of Pnma5 significantly increased after PND3 and dramatically peaked at PND21. **(C)** Pnma5 protein level was much more abundant in oocytes than in granulosa cells (GCs). **(D, E)** Westernblot showed that Pnma5was constant from the GV to MII stages during in-vitro maturation of oocytes. GV, germinal vesicle; Pro-MI, pro-metaphase I; MI, metaphase I; MII, metaphase II. n=3. **(F)** Pnma5 immunofluorescence in the mouse ovary showed that Pnma5 localized at the cortex as well as scattered within cytoplasm at different meiotic stages, while also concentrated within nucleus at GV stage. Moreover, at late MI stage when spindles translocate close to the cortex, cortical staining of Pnma5 at the translocation region was significantly lower than other cortical region. Different Z-series of confocal images of Pnma5 staining, from cortex to center, were shown below. Pnma5 in green, DNA in blue. 1pb, first polar body. Scale bar, 20 μm. **(G, H)** In both immunofluorescence and immunohistochemistry, Pnma5 localization in oocytes of antral follicles was similar to that in IVM GV oocytes. Cortical Pnma5 was arrow-pointed. In panel G Pnma5 in green, DNA in blue; in panel H, Pnma5 in brown, DNA in blue. Scale bar, 50 μm. Data are represented as mean+/- SEM.

The expression and localization pattern of Pnma5 were here examined in mouse ovaries and oocytes. RT-PCR showed that all members of the PNMA family were abundant in mouse ovaries, but Pnma5 was more prominent in oocytes than other Pnmas (Figure 1A). Western blot of Pnma5 in ovaries of different PND (post-natal day) showed that Pnma5 level in ovaries gradually increased and dramatically surged in PND 21, when the first wave of follicle maturation took place (Figure 1B). Western blot analysis showed that Pnma5 was much more abundant in oocytes than in granulosa cells (GCs) (Figure 1C) and remained constant from GV to MII stage during oocytes maturation process (Figure 1D and 1E). Immunostaining in in-vitro maturated (IVM) oocytes showed that except highly concentrated within nucleus at GV stage, Pnma5 had much stronger concentration at cortex than within cytoplasm (Figure 1F). And both immunofluorescence and immunohistochemistry in isolated ovaries and showed that Pnma5 concentrates at the cortex of oocytes (Figure 1G and 1H). From above, Pnma5 appears to be important in the maturation of oocytes and the growth of follicles.

### Pnma5 is important to the progression of meiosis

**Figure 2:**
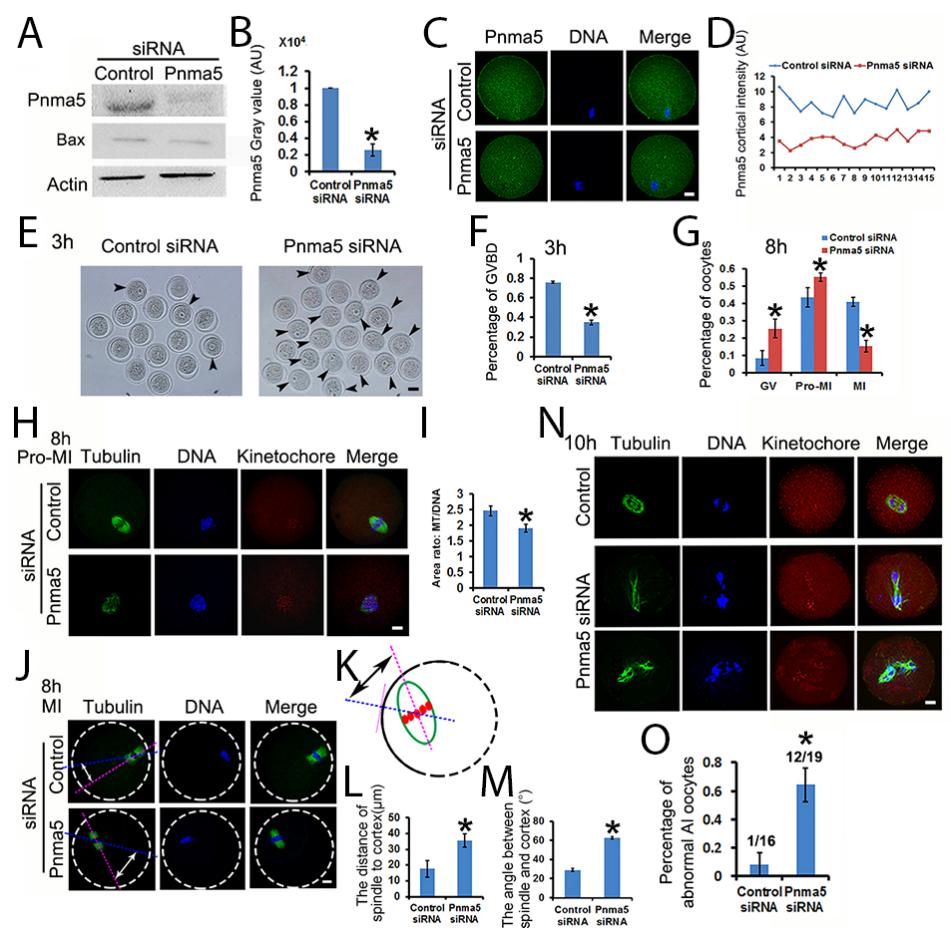
Pnma5 is important to the progression of meiosis **(A)**, **(B)**. Western blot showed that Pnma5 protein level was significantly reduced by specific siRNA. Bax level had no obvious change, which verified that siRNA transfection would not increase apoptosis. n=3 for each group. **(C), (D)** Immunofluorescence also showed that Pnma5 protein was significantly reduced by specific siRNA. And quantification of cortical Pnma5 intensity in individual oocytes showed that the knockdown fluctuation between oocytes was acceptable. Pnma5 in green, DNA in blue. **(E)**, **(F)** At 3 h of IVM, Pnma5 knockdown significantly decreased the percentage of GVBD oocytes (control vs Pnma5 knockdown, 75.81% vs 34.89%). GV oocytes were arrow-pointed. Scale bar, 50 μm; n=3 for each group. **(G)** At 8 h of IVM, there were significantly more GV oocytes (control vs Pnma5 knockdown, 8.40% vs 25.46%) and pro-MI oocytes (control vs Pnma5 knockdown, 43.51% vs 55.19%) and less MI oocytes (control vs Pnma5 knockdown, 40.90% vs 15.33%) in Pnma5-knockdown group, n=3 for each group. **(H)**, **(I)** At 8 h of IVM, the chromosomes in Pnma5-knockdown pro-MI oocytes were significantly less congressed than in controls as manifested by the area ratio of spindle microtubules/DNA (control vs Pnma5 knockdown, 2.46 vs 1.90). n=11 for each group. **(J-M)** We also measured the shortest distance from the center of spindles to the closest cortex (control vs Pnma5 knockdown,17.77 μm vs 35.76 μm) and the angle between spindle long axis and the shortest line (which correspond to the shortest distance above) of spindle (control vs Pnma5 knockdown, 28.98 ° vs 62.56 °), results showed that Pnma5 knockdown significantly increased the relative distance and angle of MI spindle to cortex. n=21 for control, n=32 for Pnma5 knockdown. **(N)**, **(O)** At 10 h of IVM, significantly more Pnma5-knockdown anaphase oocytes had obvious lagging chromosomes than control oocytes (control vs Pnma5 knockdown, 8.3% vs 64.29%). n=3 for each group, numbers above each column in panel (O) denote number of anaphase oocytes with lagging chromosomes / number of total anaphase oocytes examined. Tubulin in green, DNA in blue, kinetochores in red. Scale bars for other panels, 20 μm. Data are represented as mean+/- SEM. Significant differences are labeled with an asterisk (^*^).

Next, Pnma5 was knocked down at the protein level with specific siRNAs, and the effects on meiosis were examined. Both Western blot and immunostaining showed that Pnma5 had been eliminated efficiently (Figure 2A-2C). Meanwhile, the apoptosis-related marker Bax level had no obvious change, which verified that siRNA transfection would not increase apoptosis (Figure 2A). And quantification of cortical Pnma5 intensity in individual oocytes showed that the knockdown fluctuation between oocytes was acceptable (Figure 2D). At 3 h of IVM, Pnma5 knockdown significantly decreased the percentage of GVBD oocytes (control vs Pnma5 knockdown, 75.81% vs 34.89%) (Figure 2E and 2F). At 8 h of IVM, there were significantly more oocytes at GV (control vs Pnma5 knockdown, 8.40% vs 25.46%) and pro-MI stage (control vs Pnma5 knockdown, 43.51% vs 55.19%) and less oocytes at MI stage (control vs Pnma5 knockdown, 40.90% vs 15.33%) after Pnma5 knockdown (Figure 2G). Moreover, the chromosomes in Pnma5-knockdown pro-MI oocytes were significantly less congressed than in control as manifested by the area ratio of spindle microtubules / DNA (control vs Pnma5 knockdown, 2.46 vs 1.90) (Figure 2H and 2I). We also measured the shortest distance from the center of spindles to the closest cortex (control vs Pnma5 knockdown, 17.77 μm vs 35.76 μm) (Figure 2J-2L) and the angle between spindle long axis and the shortest line (which correspond to the shortest distance above) of spindle (control vs Pnma5 knockdown, 28.98 ° vs 62.56 °) (Figure 2J,2K and 2M), results showed that Pnma5 knockdown significantly increased the relative distance and angle of MI spindle to cortex, indicating that the MI spindle translocation was significantly delayed after Pnma5 knockdown (Figure 2J-2M). At 10 h of IVM, significantly more Pnma5-knockdown anaphase oocytes had obvious lagging chromosomes than control oocytes (control vs Pnma5 knockdown, 8.3% vs 64.29%) (Figure 2N and 2O).

### Pnma5 is important to oocyte maturation and normal fertilization

**Figure 3:**
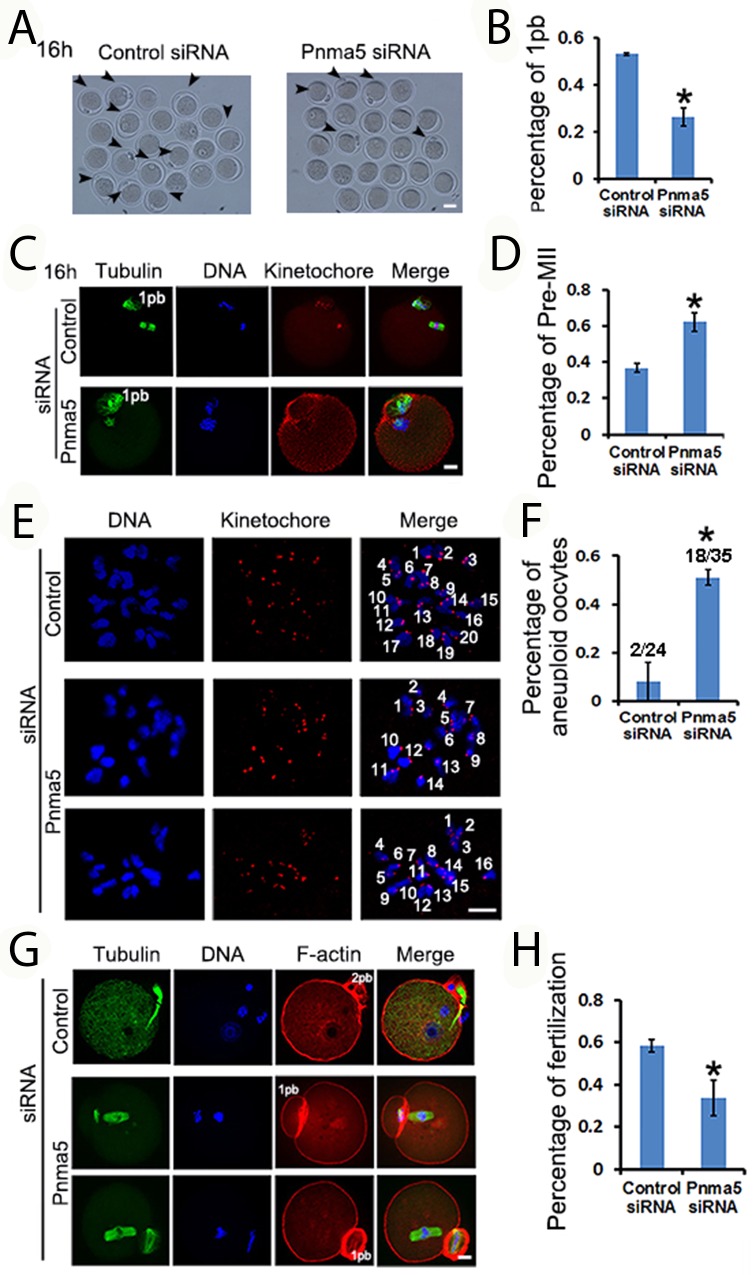
Pnma5 is important to oocyte maturation and normalfertilization **(A-D)** At 16 h of IVM, Pnma5 knockdown significantly reduced the relative amount of first polar body extrusion in oocytes. And there were significantly more MII oocytes with non-congressed chromatids in the Pnma5 knockdown group than in other groups, these were here called “Pre-MII oocytes”. Oocytes with first polar body were arrow-pointed. Tubulin in green, DNA in blue, kinetochores in red. n=3 for each group. **(E, F)** Chromosome spreading experiment showed significantly greater proportions of MII oocytes with aneuploidy in the Pnma5-knockdown group than in the control group (control vs Pnma5 knockdown, 8.2% vs 51.3%), n=3 for each group, numbers above each column in panel F denote number of MII oocytes with aneuploidy / number of total MII oocytes examined. DNA in blue, kinetochores in red. **(G)**, **(H)** *in vitro* fertilization (IVF) results showed Pnma5 knockdown to be associated with a significantly lower fertilization rate than in controls (control vs Pnma5 knockdown, 58.38% vs 33.93%). Tubulin in green, DNA in blue, F-actin in red. n=3 for each group. 1pb, first polar body; 2pb, second polar body. Scale bars, 20 μm. Data are represented as mean+/- SEM. Significant differences are labeled with an asterisk (^*^).

Next the role of Pnma5 in oocyte maturation and fertilization was evaluated. At 16 h of IVM, Pnma5 knockdown significantly reduced the percentage of first polar body extrusion in oocytes (control vs Pnma5 knockdown, 53.23% vs 26.41%) (Figure 3A and 3B). And there were significantly more MII oocytes with non-congressed chromatids in the Pnma5-knockdown group than in control, these were here called “Pre-MII oocytes” (control vs Pnma5 knockdown, 36.84% vs 62.20%) (Figure 3C and 3D). A chromosome spreading experiment indicated significantly greater proportions of MII oocytes with aneuploidy in the Pnma5-knockdown group than in the control group (control vs Pnma5 knockdown, 8.2% vs 51.3%) (Figure 3E and 3F). *In-vitro* fertilization (IVF) results showed Pnma5 knockdown was associated with a significantly lower fertilization rate than in controls (control vs Pnma5 knockdown, 58.38% vs 33.93%) (Figure 3G and 3H).

### Pnma5 is required for the stability of spindle microtubules and F-actin

**Figure 4:**
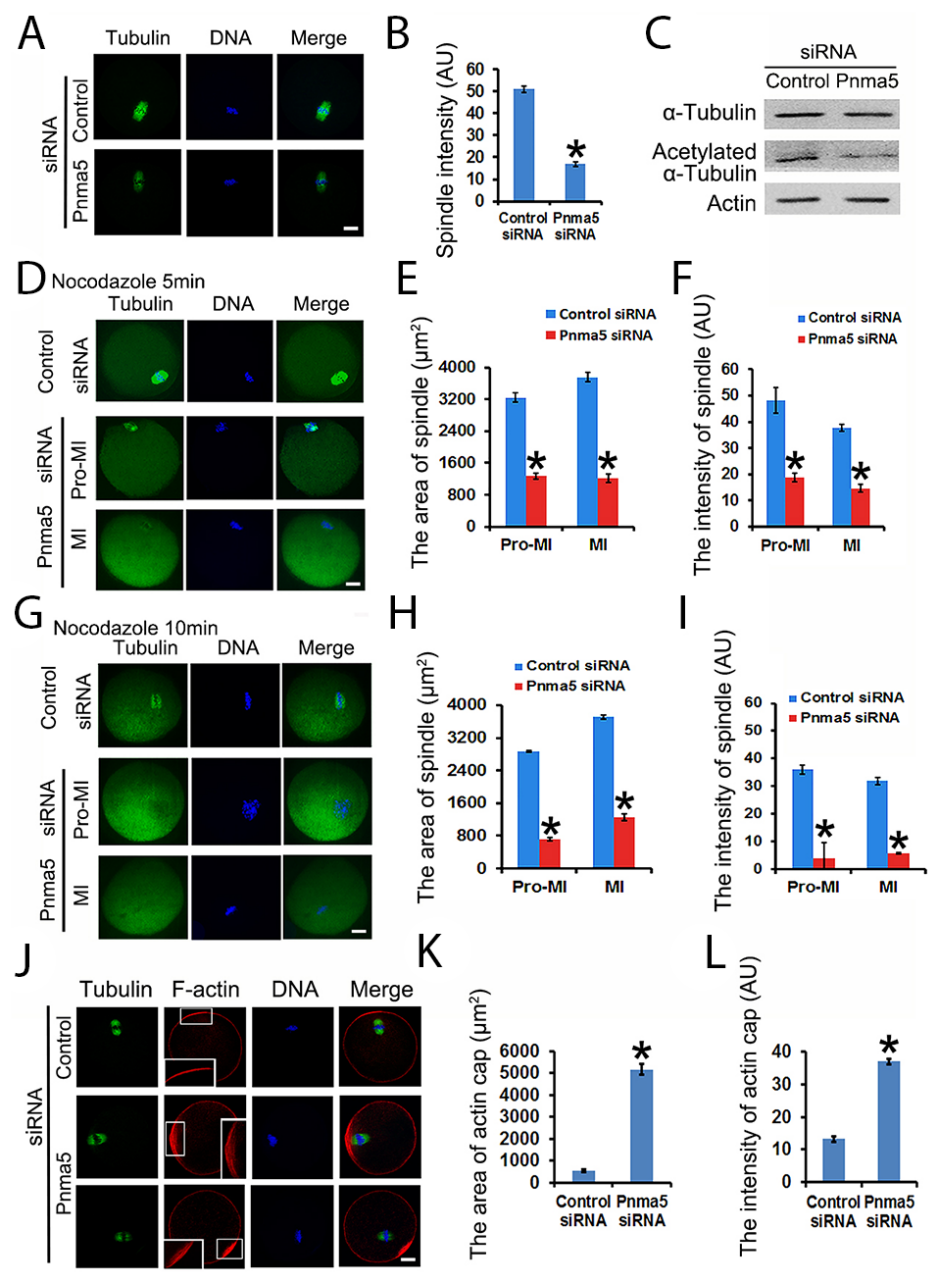
Pnma5 is required for spindle microtubule and F-actin stability **(A)**, **(B)** Immunofluorescence showed that Pnma5 knockdown significantly reduced microtubule intensity of MI spindles. n=34 for control, n=40 for Pnma5 knockdown group. **(C)** Western blot showed that Pnma5 knockdown significantly diminished acetylated α-tubulin level. **(D-F)** After 5 min of depolymerization, the area and fluorescence intensity of spindle microtubules in Pnma5-knockdown group were significantly smaller than in controls for both pro-MI and MI stage oocytes (control vs Pnma5 knockdown, the area of spindle in pro-MI oocytes, 3246.33 μm^2^ vs 1270.14 μm^2^; in MI oocytes, 3756.93μm^2^ vs 1214.5μm^2^; the intensity of spindle microtubules in pro-MI oocytes, 48.07 vs 18.88; in MI oocytes, 37.77 vs 14.62). n=16 for control, n=20 for pnma5 knockdown group. **(G-I)** After 10 min of depolymerization, the differences were even more significant (control vs Pnma5 knockdown, the area of spindle in pro-MI oocytes, 2875.19 μm^2^ vs 712.72 μm^2^; in MI oocytes, 3714.27 μm^2^ vs 1248.54 μm^2^; the intensity of spindle in pro-MI oocytes, 36.04 vs 4.01; in MI oocytes, 31.91 vs 5.68). n=15 for control, n=18 for Pnma5 knockdown group. (I), **(K)** Thearea of the actin cap region (control vs Pnma5 knockdown, 530.14 μm^2^ vs 5157.61 μm^2^) significantly increased after Pnma5 knockdown. Actin cap regions were zoomed to show the organization of F-actin. Pnma5 knockdown appeared to significantly disrupt F-actin organization at actin cap regions. **(J)**, **(L)** The fluorescence intensity of F-actin within actin cap region significantly increased after Pnma5 knockdown (control vs Pnma5 knockdown, 11.42 vs 38.3). Tubulin in green, DNA in blue, F-actin in red. Scale bars, 20 μm. Data are represented as mean+/- SEM. Significant differences are labeled with an asterisk (^*^).

Because Pnma5 knockdown significantly affects meiotic progression, next we want to further investigate whether or how Pnma5 knockdown could affect the spindles. First, Pnma5 knockdown significantly reduced microtubule intensity of MI spindles (Figure 4A and 4B). Next results showed that there was significantly less acetylated α-tubulin in the Pnma5-knockdown group than in control, while the total tubulin level remained unchanged (Figure 4C), suggesting that the decrease in the intensity of spindle microtubules was mainly due to the decreased acetylated α-tubulin. For further verification, nocodazole was used to depolymerize microtubules for 5 or 10 min and microtubule stability was examined. Results showed that, after 5 min of depolymerization, the area or fluorescence intensity of spindle microtubules in Pnma5-knockdown group were significantly smaller or lower than in controls for both pro-MI and MI stage oocytes (control vs Pnma5 knockdown, the area of spindle in pro-MI oocytes, 3246.33 μm^2^ vs 1270.14 μm^2^; in MI oocytes, 3756.93μm^2^ vs 1214.5μm^2^; the intensity of spindle microtubules in pro-MI oocytes, 48.07 vs 18.88; in MI oocytes, 37.77 vs 14.62) (Figure 4d–4f). After 10 min of depolymerization, the differences were even more significant (control vs Pnma5 knockdown, the area of spindle in pro-MI oocytes, 2875.19 μm^2^ vs 712.72 μm^2^; in MI oocytes, 3714.27 μm^2^ vs 1248.54 μm^2^; the intensity of spindle in pro-MI oocytes, 36.04 vs 4.01; in MI oocytes, 31.91 vs 5.68) (Figure 4G-4I). These results suggest that Pnma5 promotes spindle organization by stabilizing spindle microtubules.

Since Pnma5 knockdown significantly delayed normal MI spindle translocation, which is largely dependent on the normal function of actin cap [28, 29], we then want to examine whether or how Pnma5 knockdown could affect the organization of actin cap. Immunostaining showed that compared with control, Pnma5 knockdown significantly increased the area (control vs Pnma5 knockdown, 530.14 μm^2^ vs 5157.61 μm^2^) (Figure 4J and 4K) and fluorescence intensity (control vs Pnma5 knockdown, 11.42 vs 18.3) (Figure 4J and 4L) of F-actin at actin cap region. In addition, the F-actin in the Pnma5-knockdown oocytes at the actin cap region became obviously disorganized (Figure 4J, enlarged insert images). These results indicate that the normal function of actin cap might be disturbed by Pnma5 knockdown.

### Pnma5 is required for mitochondria dynamics and function

**Figure 5:**
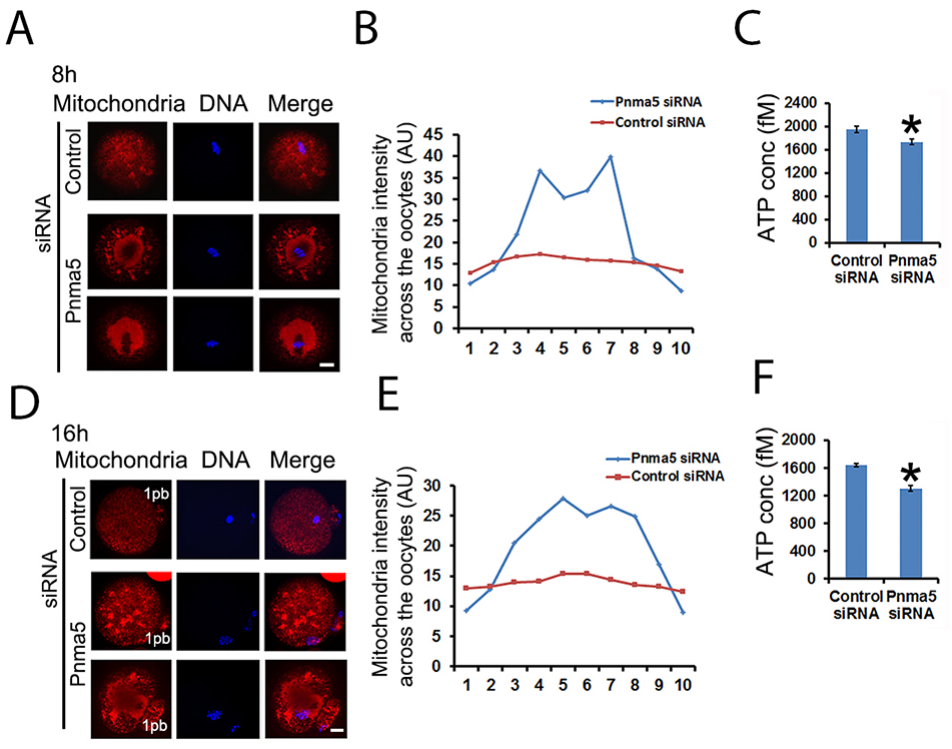
Pnma5 is required for mitochondria dynamics and function **(A)**, **(B)** and **(D, E)** At both 8 h (A) and 16 h (D) of IVM, the mitochondria were dispersed as small particles throughout the cytoplasm of control oocytes and had moderately higher concentration around MI spindles; while in the cytoplasm of Pnma5-knockdown oocytes, the mitochondria formed many big aggregates around spindles. Next oocytes were subdivided into ten parallel sub-regions and careful measurement of the mitochondria intensity of each sub-regions also showed that mitochondria intensity seemed much higher in the center than at cortex (B, E). **(C)** and **(F)** Accordingly, ATP levels were significantly lower in Pnma5-knockdown oocytes than in controls. Mitochondria in red, DNA in blue. 1pb, first polar body. n=3 for each group. Scale bars, 20 μm. Data are represented as mean+/- SEM. Significant differences are labeled with an asterisk (^*^).

Mitochondrial dynamics is key to ATP generation and thereby essential to normal meiosis. So we wanted to see whether Pnma5 is important for normal mitochondria dynamics. Mitotracker staining revealed that, at both 8h (Figure 5A and 5B) or 16 h (Figure 5D and 5E) of IVM, the mitochondria were dispersed as small particles throughout the cytoplasm of control oocytes and had moderately higher concentration around spindles; while in the cytoplasm of Pnma5-knockdown oocytes, the mitochondria formed many big aggregates around spindles. And careful measurement of the mitochondria intensity of each sub-regions also showed that mitochondria intensity seemed much higher in the center than at cortex. (Figure 5B and 5E). Accordingly, ATP levels were significantly lower in Pnma5-knockdown oocytes than in controls (Figure 5C and 5F). These results showed that Pnma5 is important for normal mitochondria dynamics and ATP generation.

### The activity of Pnma5 is regulated by phosphorylation

**Figure 6:**
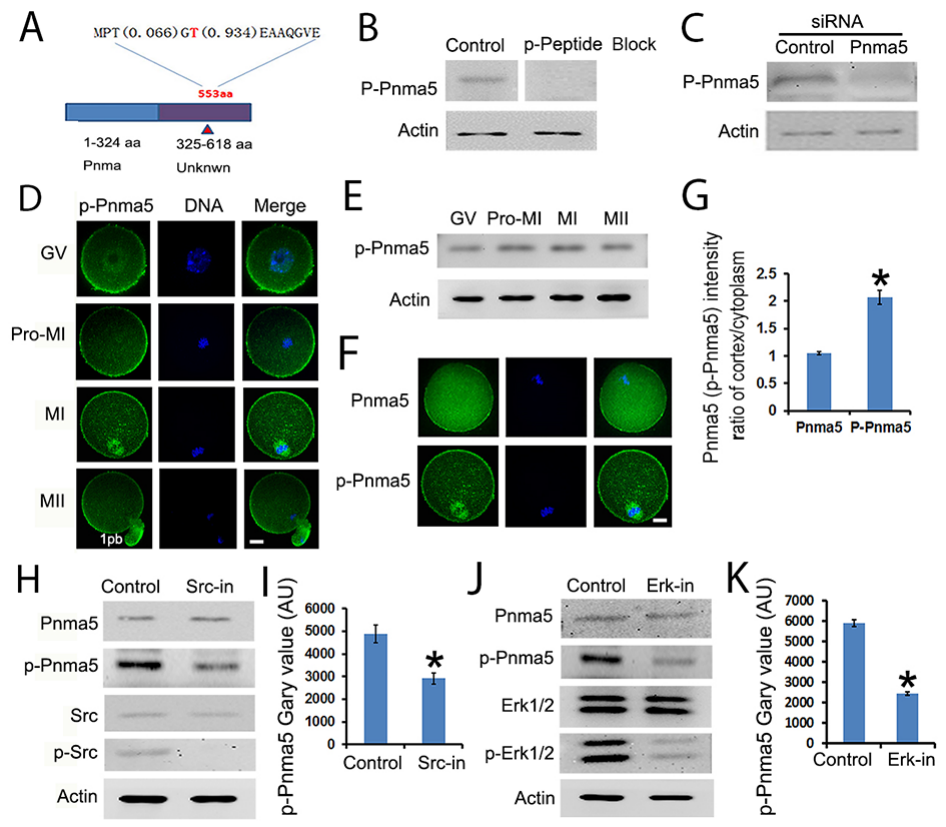
The activity of Pnma5 is regulated by phosphorylation **(A)** Through phospho-peptide enrichment and LC-MS after IP, an attractive site was identified at Thr553 (phosphorylation possibility 93.4%) in the non-defined domain of Pnma5 and a phospho antibody specific to Thr553 (p-Pnma5) was generated. **(B, C)** The specificity of p-Pnma5 antibody was confirmed using both phospho peptide blocking-blot (B) and siRNA knockdown-blot **(C)**. **(D)** Immunolocalization showed that phosphorylated Pnma5 (p-Pnma5) had significantly stronger concentration both at cortical regions from GV to MII and within spindle regions at MI oocytes. **(E)** p-Pnma5 remained constant during oocytes maturation process through Western Blot. **(F)** and **(G)** Fluorescence intensity quantification of cortical t-Pnma5 or p-Pnma5 in MI oocytes showed that the cortical intensity of p-Pnma5 was much higher than t-Pnma5 (t-Pnma5 vs p-Pnma5, 1.05 vs 2.07). n=35 for t-Pnma5, n=38 for p-Pnma5 knockdown group. **(H)** and **(I)** Western blot showed that inhibition of Src phosphorylation significantly decreased p-Pnma5, n=3 for each group. **(J)** and **(K)** Western blot showed that inhibition of Erk 1/2 phosphorylation also significantly decreased p-Pnma5. n=3 for each group. Pnma5 or p-Pnma5 in green, DNA in blue. 1pb, first polar body. Scale bars, 20 μm. Data are represented as mean+/- SEM. Significant differences are labeled with an asterisk (^*^).

To uncover the molecular mechanism by which Pnma5 functions, the effect of phosphorylation, the most common means of modulating protein activity, on the regulation of Pnma5 activity was here investigated. Through phospho-peptide enrichment and LC-MS after IP, an attractive site was identified at Thr553 (phosphorylation possibility 93.4%) in the non-defined domain of Pnma5 (Figure 6A) and a phospho antibody specific to Thr553 was generated. Its specificity was confirmed using a phospho peptide blocking-blot and Pnma5 knockdown-blot (Figure 6B and 6C). Western blot showed that p-Pnma5 remained constant during oocytes maturation process (Figure 6E). Immunostaining showed that phosphorylated Pnma5 (p-Pnma5) appears to concentrate at cortex more than total Pnma5 (t-Pnma5) (Figure 6D), and intensity quantification of t-Pnma5 or p-Pnma5 in MI oocytes did showed that the cortical intensity of p-Pnma5 was much higher than t-Pnma5 (t-Pnma5 vs p-Pnma5,1.05 vs 2.07) (Figure 6F and 6G). And particularly, p-Pnma5 also highly concentrated within MI spindles (Figure 6D). These results indicate that Pnma5 phosphorylation might be important for its meiotic function.

To investigate how Pnma5 facilitates meiotic progression, several potential Pnma5-interacting proteins were characterized through IP-maldi and a pathway model was created. In this model, Src→Erk 1/2 might be the upstream of Pnma5. Western blot analysis did show that inhibition of either Src or Erk 1/2 phosphorylation could significantly decrease p-Pnma5 (Figure 6H-6K). These results indicate that Pnma5 is activated through phosphorylation by Src→Erk 1/2 pathway.

### Pnma5 phosphorylation regulates meiosis through the Akt→Gsk3β pathway

**Figure 7:**
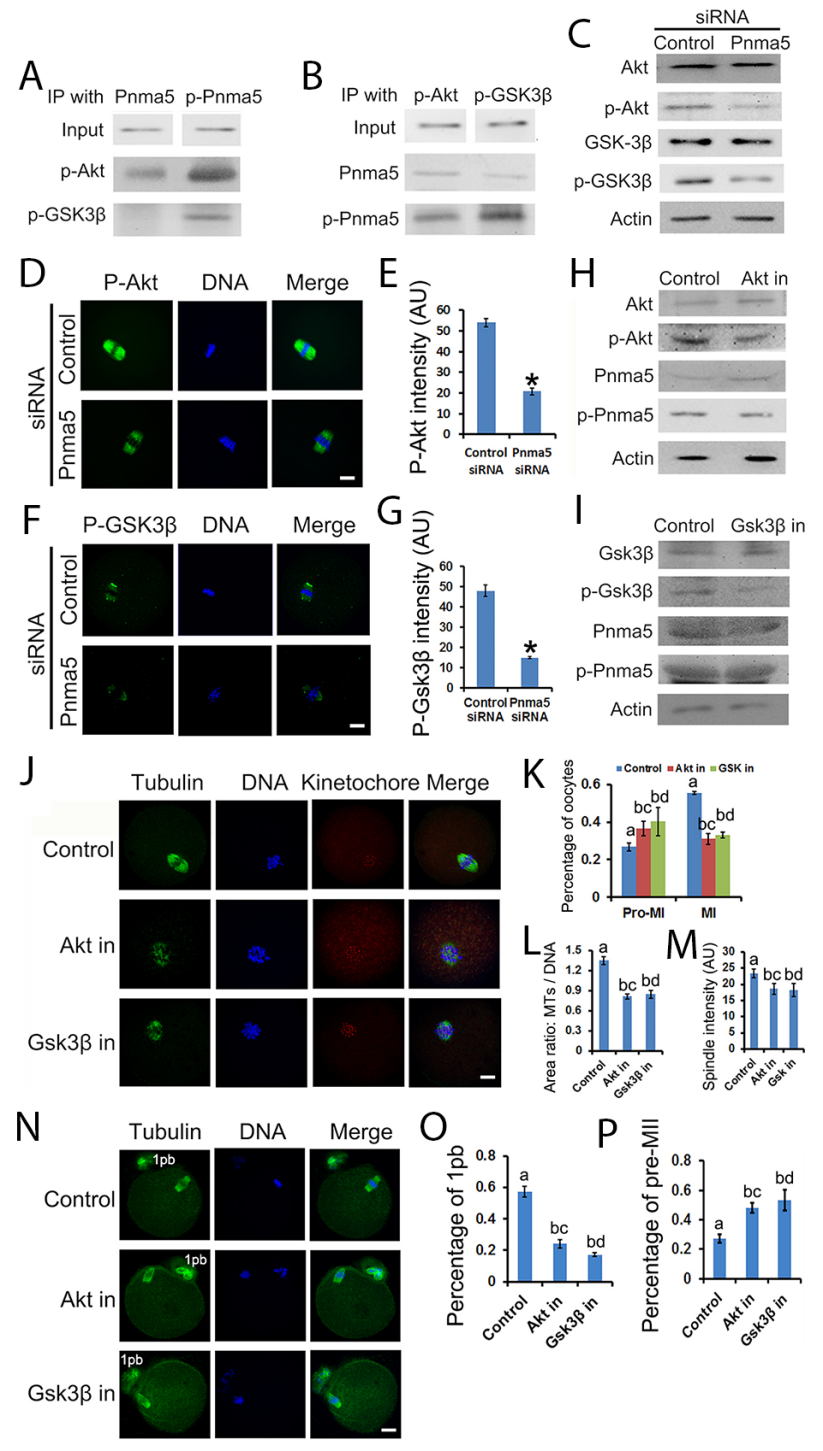
Pnma5 phosphorylation regulates meiosis through the Akt→Gsk3β pathway **(A)**, **(B)** Co-immunoprecipitation and blot showed that both p-Akt and p-Gsk3β interacted well with p-Pnma5 but much less with Pnma5. In A, Pnma5 or p-Pnma5 antibody were used as baits and detected by themselves, p-Akt or p-Gsk3β antibodies. In B, p-Akt or p-Gsk3β antibodies were used as baits and detected by themselves, Pnma5 or p-Pnma5 antibodies. **(C)** Western blot showed that Pnma5 knockdown significantly diminished protein level of p-Akt or or p-Gsk3β. **(D)**, **(E)** Consistent with **(C)** immunofluorescence showed that Pnma5 knockdown significantly reduced p-Akt intensity within spindles (Control vs Pnma5 knockdown, 53.96 vs 20.77). n=32 for control, n=42 for pnma5 knockdown group. **(F)**, **(G)** Consistent with **(C)** Immunofluorescence showed that Pnma5 knockdown significantly reduced p-Gsk3β intensity within spindles (Control vs Pnma5 knockdown, 48.03 vs 15.03). n=34 for control, n=38 for pnma5 knockdown group. **(H)**, **(I)** We found that expression of Pnma5 and phosphor-Pnma5 had no obvious change after Gsk3β and Akt were inhibited. **(J)**, **(K)** At 8h of IVM after Gsk3β inhibition (Gsk3β in) or Akt inhibition (Akt in), there were significantly more pro-MI oocytes (control vs Akt in vs Gsk3β in, 26.75% vs 36.58% vs 40.21%) and less MI oocytes (control vs Akt in vs Gsk3β in, 55.5% vs 31.04% vs 33.05%). n=3 for each group. **(J)**, **(L)** The ratio of microtubule area/DNA area (control vs Akt in vs Gsk3β in,1.35 vs 0.82 vs 0.85) significantly decreased after Akt or Gsk3β inhibition. n=61 for control, n=60 for Akt in, n=67 for Gsk3β in. **(J)**, **(M)** The fluorscence intensity of spindles (control vs Akt in vs Gsk3β in,23.31% vs 18.59% vs 18.27%) significantly decreased after Akt or Gsk3β inhibition. n=61 for control, n=60 for Akt in, n=67 for Gsk3β in. **(N-P)** At 16 h of IVM, pnma5 knockdown significantly reduced the percentage of first polar body extrusion in oocytes (control vs Akt in vs Gsk3β in,57.26% vs 24.11% vs 17.21%) and increased the percentage of Pre-MII oocytes (control vs Akt in vs Gsk3β in,27.27% vs 48.10% vs 53.18%). n=3 for each group. For D and F, p-Akt or p-Gsk3β in green, DNA in blue. For J and N, Tubulin in green, DNA in blue, kinetochores in red. 1pb, first polar body. Scale bars, 20 μm. Data are represented as mean+/- SEM. For comparison between two groups, significant differences are labeled with an asterisk (^*^); For comparison among three groups, different numbers above the columns mean significant differences.

The pathway model also suggest that p-Pnma5 interacts with and activates Akt→Gsk3β to regulate meiosis. In support of this, firstly co-IP and blot showed that both p-Akt and p-Gsk3β co-IPed well with p-Pnma5 but much less with Pnma5 (Figure 7A and 7B); secondly both immunostaining and western blot showed that Pnma5 knockdown significantly decreased the levels of p-Akt (p-Akt fluorescence intensity, control vs Pnma5 knockdown, 53.96 vs 20.77) (Figure 7C, 7D and 7E) and p-Gsk3β (p-Gsk3β fluorescence intensity, control vs Pnma5 knockdown, 48.03 vs 15.03) (Figure 7C, 7F and 7G). For further verification, we used Gsk3β or Akt inhibitor to dispose the oocytes and examined the t-Pnma5 and p-Pnma5 expression. We found no obvious change for both t-Pnma5 and p-Pnma5 after Gsk3β or Akt were inhibited (Figure 7H and 7I), indicating that Pnma5 did function upstream of Gsk3β and Akt.

Since the evidence above suggested that Akt→Gsk3β might be the downstream of Pnma5, we want to further verify whether the dysfunction of Gsk3β or Akt could cause similar meiotic phenotypes. Results showed that, at 8h of IVM after Gsk3β inhibition (GSK3β in) or Akt inhibition (Akt in), there were significantly more oocytes at pro-MI (control vs Akt in vs GSK3β in, 26.75% vs 36.58% vs 40.21%) stage and less at MI (control vs Akt in vs Gsk3β in, 55.5% vs 31.04% vs 33.05%) stage (Figure 7J and 7K). Moreover, the chromosomes in Akt-in or Gsk3β-in pro-MI oocytes were significantly less congressed than in controls as manifested by microtubule area/DNA area (control vs Akt in vs Gsk3β in,1.35 vs 0.82 vs 0.85) (Figure 7J and 7L); and the fluorescence intensity of spindle microtubules (control vs Akt in vs Gsk3β in, 23.31% vs 18.59% vs 18.27%) also significantly decreased (Figure 7J and 7M). At 16 h of IVM, Akt-in or Gsk3β-in significantly reduced the percentage of first polar body extrusion in oocytes (control vs Akt in vs Gsk3β in,57.26% vs 24.11% vs 17.21%) (Figure 7N and 7O) and increased the percentage of Pre-MII oocytes (control vs Akt in vs Gsk3β in, 27.27% vs 48.10% vs 53.18%) (Figure 7N and 7P). All these results appear to be quite similar to the phenotypes in Pnma5-knockdown oocytes, which further support our model.

## DISCUSSION

Oocytes is a unique type of cells for reproduction. They store many special fertility factors for the accomplishment of oocyte maturation and zygote development. Some of these factors are expressed and functionally utilized only in ovaries and oocytes. For example, growth factors BMP15 and GDF9 secreted by oocytes promote the proliferation of granulosa cells, folliculogenesis and ovary development [30–33]. The rest of these factors belong to certain protein families but some members are selected to be much more prominently expressed in ovaries and oocytes than other family members and thereby might be also functionally important only in ovaries and oocytes. For example, PADI6 is uniquely localized in oocytes and embryos, where it plays important roles in early embryonic development and female fertility [34, 35]. The object of the current study, Pnma5, is part of the paraneoplastic Ma (PNMA) family, which consists of Pnma1, Pnma2, Pnma3, Pnma4 (Moap1), Pnma5, and Pnma6A. Although it shares a conserved PNMA domain with the other Pnmas, the expression pattern, cellular localization and correlation with diseases is very different [9–23]. Pnma5 was thought to be a potential female fertility factor but no any related functional reports yet [24]. As indicated in the current study, Pnma5 is much more prominent in oocytes than in GCs and is the most abundant among PNMAs in oocytes, suggesting that compared with the others, Pnma5 is the selected Pnma family member particularly important in oocytes. There is considerable functional and regulatory evidences to support this.

First, Pnma5 knockdown was found to cause significant meiotic defects, which was manifested mainly through delayed meiotic resumption, disturbed meiosis I, abnormal anaphase I with lagging chromosomes. Pnma5 knockdown also significantly decreased the level of acetylated tubulin, a major form of stable microtubules within spindles, which were also closely related to the meiotic defects. Notably, Pnma5 knockdown significantly affected the translocation of MI spindles and the organization of F-actin at actin cap region. Considering p-Pnma5 specifically highly concentrated within MI spindles, p-Pnma5 might be directly responsible for the normal organization & function of actin cap.

Secondly, Pnma5 knockdown dramatically increased abnormal MII oocytes with less congressed chromosomes. Since abnormal chromosome congression is an important form of cytoplasmic immaturation and has been thought to cause decreased fertilization rate [36, 37], so probably it is one of the major factors causing abnormal fertilization in Pnma5-knockdown oocytes. And Pnma5 knockdown also dramatically disrupted mitochondrial distribution and significantly decreased ATP levels in maturated MII oocytes. Since mitochondria malfunction in oocytes has also been shown to reduce fertilization [38, 39], so probably it is also a major origin of abnormal fertilization in Pnma5-depleted oocytes.

To investigate how Pnma5 exert its roles, its interacting proteins were characterized and a working model was created and then has been well verified. Firstly, our results showed that Pnma5 phosphorylation is controlled by upstream phosphorylation of Src→Erk1/2, since it's well-known that Src→Erk1/2 is activatied by phosphorylation, so probably the phosphorylated Pnma5 is the active form of total Pnma5. Secondly, our results also showed that Pnma5 could phosphorylate downstream Akt→Gsk3β. Akt has been shown to regulate oocyte meiosis, and it can be targeted to mitochondria and regulate phosphorylation of PDK1 that is essential to mitochondrial function [40–42]. Gsk3β has been shown to regulated spindle stability and meiosis through phosphorylation [43]. And Gsk3β has also been shown to regulate actin-dependent dynamics of focal adhesion or growth cone [44, 45], suggesting that Pnma5 might affect actin cap through Gsk3β. So Pnma5 probably function in meiosis by controlling the phosphorylation of these two important kinases. Actually, Pnma1 had also been shown to activate Akt→Gsk3β pathway [13].

In conclusion, our study indicated that Pnma5 is the selected Pnma family protein important to meiotic progression. The signal pathway for Pnma5 appears to be Src→Erk1/2→Pnma5→Akt→Gsk3β, where Pnma5 is at a pivotal position. This is the first study of the function and regulation of Pnma5 in oocytes. Further investigation is needed to confirm the specifics of this pathway.

## MATERIALS AND METHODS

### General chemicals & reagents and animals

Chemicals & reagents were obtained from Sigma unless otherwise stated. 3-4 week-old female ICR mice used in this study were from Vitalriver experimental animal technical co, LTD of Beijing. All animal experiments were approved by the Animal Care and Use Committee of Nanjing Medical University and were performed in accordance with institutional guidelines.

### Antibodies & inhibitors

Mouse monoclonal anti-β-actin (Cat#: A5316-100), mouse monoclonal anti-α-tubulin antibody (Cat#: sc-8035), mouse monoclonal anti-β-tubulin antibody (Cat#: sc-5274), mouse monoclonal anti acetylated α-tubulin antibody (sc-23950) were purchased from Santa Cruz (Dallas, Texas, USA). Human anti-centromere CREST antibody (Cat#:15–234) was purchased from Antibodies Incorporated (Davis, California, USA). Cy2-conjugated donkey anti-Rabbit IgG (Code:711-225-152), rhodamine(TRITC)-conjugated donkey anti-Rabbit IgG (Code:711-025-152), and 647-conjugated donkey anti-Human IgG (Code:709-605-149) were purchased from Jackson ImmunoResearch Laboratory (West Grove, Pennsylvania, USA). Horseradish Peroxidase (HRP)-conjugated goat anti rabbit IgG and HRP-conjugated goat anti mouse IgG were purchased from Vazyme (Nanjing, Jiangsu, China). Rabbit anti Bax polyclonal antibody (Cat#:23931-1-AP) was purchased from Proteintech (Rosemont, Illinois, USA). Rabbit anti Phospho-Akt (Ser473) (193H12) antibody (Cat#:4058) was purchased from Cell Signaling (Danvers, Massachusetts, USA). Mouse Monoclonal Antibody p-Erk1/2 (M9692) was purchased from Sigma (St.Louis, MO, USA). Rabbit anti p-Gsk3β (GTX50090) was purchased from GeneTex (Alton Parkway Irvine, USA). Rabbit anti p-PNMA5 was produced by Zhong Ding Biotechnique, Ltd. (Nanjing, Jiangsu, China). Akt protein inhibitor (MK-2206 2Hcl), Gsk3β protein inhibitor (IM-12), Src protein inhibitor (KX2-391) and Erk1/2 protein inhibitor (SCH772984) were purchased from Selleck (Houston, Texas, USA).

### Oocytes collection and culture

Immature oocytes arrested in prophase I (GV oocytes) were obtained from the ovaries of 3-4 week-old ICR female mice in natural estrus. The mice were first euthanatized with CO2 and then sacrificed by cervical dislocation, and ovaries were isolated and placed in operation medium (Hepes) with 2.5 nM milrinone and 10% fetal bovine serum (FBS) (Gibco). Oocytes were released from the ovary by puncturing the follicles with a hypodermic needle. Cumulus cells were washed off the cumulus-oocyte complexes and every 50 Isolated denuded oocytes were placed in 100 ul droplets of culture medium under mineral oil (Sigma) in plastic dishes (BD). The culture medium was MEM+ containing 20% FBS (MEM+ means MEM with 0.01 mM EDTA, 0.23 mM Na-pyruvate, 0.2 mM Penicillin/Streptomycin, 3 mg/ml BSA). Oocytes were cultured at 37.0 °C, 5% O_2_, 5% CO_2_ in humidified atmosphere. Prior to *in vitro* maturation (IVM ), all culture medium include 2.5 nM milrinone to prevent resumption of meiosis.

### SiRNA production and transfection

Sequences of all DNA templates for siRNA production are listed in Table 1. The sequence of control templates is a mock sequence that does not specifically bind to any mRNA from the mouse genome. DNA templates against four different coding for DNA sequence (CDS) regions of Pnma5 siRNA were designed online through BLOCK-iT™ RNAi Designer (http://rnaidesigner.invitrogen.com/rnaiexpress/s) with some modification. Sequence specificity was verified through a blast homology search.

**Table 1:** DNA templates for siRNA production

Target Site	DNA templates
Pnma5 CDS 202-224^1^	Oligo1: GGATCCTAATACGACTCACTATAGAACTGCCTGAAGTTGTTGATTA2
	Oligo2: AATAATCAACAACTTCAGGCAGTTCTATAGTGAGTCGTATTAGGATCC^2^
	Oligo3: GGATCCTAATACGACTCACTATATAATCAACAACTTCAGGCAGTTC2
	Oligo4: AAGAACTGCCTGAAGTTGTTGATTATATAGTGAGTCGTATTAGGATCC^2^
Pnma5 CDS 1110-1132^1^	Oligo1: GGATCCTAATACGACTCACTATAGACCACAGGAGAGGAGATGACAT2
	Oligo2: AAATGTCATCTCCTCTCCTGTGGTCTATAGTGAGTCGTATTAGGATCC^2^
	Oligo3: GGATCCTAATACGACTCACTATAATGTCATCTCCTCTCCTGTGGTC2
	Oligo4: AAGACCACAGGAGAGGAGATGACATTATAGTGAGTCGTATTAGGATCC^2^
Pnma5 CDS 1465–1485^1^	Oligo1: GGATCCTAATACGACTCACTATA GATCCCAACAAGCAAGAACAA2
	Oligo2: AATTGTTCTTGCTTGTTGGGATCTATAGTGAGTCGTATTAGGATCC^2^
	Oligo3: GGATCCTAATACGACTCACTATATTGTTCTTGCTTGTTGGGATC2
	Oligo4: AA GATCCCAACAAGCAAGAACAATATAGTGAGTCGTATTAGGATCC^2^
Pnma5 CDS 1621-1641^1^	Oligo1: GGATCCTAATACGACTCACTATA GAGGATGGGTGTTCTGAGCTA2
	Oligo2: AATAGCTCAGAACACCCATCCTCTATAGTGAGTCGTATTAGGATCC^2^
	Oligo3: GGATCCTAATACGACTCACTATATAGCTCAGAACACCCATCCTC2
	Oligo4: AA GAGGATGGGTGTTCTGAGCTATATAGTGAGTCGTATTAGGATCC^2^
Control^3^	Oligo1: GGATCCTAATACGACTCACTATAGACCTACGCCACCAATTTCGT^2^
	Oligo2: AAACGAAATTGGTGGCGTAGGTCTATAGTGAGTCGTATTAGGATCC^2^
	Oligo3: GGATCCTAATACGACTCACTATAACGAAATTGGTGGCGTAGGTC^2^
	Oligo4: AAGACCTACGCCACCAATTTCGTTATAGTGAGTCGTATTAGGATCC^2^

^1^ The numbers are the starting and ending position of the target sites in Pnma5 CDS (NM_001100461.3 in NCBI).

^2^ Two pairs of DNA oligos are needed for each double-stand siRNA. Oligo 2 is complementary with Oligo 1 except an “AA” overhang at 5'; Oligo 3 is complementary with oligo 4 except an “AA” overhang at 5'. In each oligo, gene-specific sequences are underlined, other sequences are for recognition and binding by T7 RNA polymerase.

^3^ Control siRNA does not target to any mRNA sequence in mouse.

SiRNAs were produced using the T7 RiboMAX^™^ Express RNAi System (Promega) according to the manufacturer’s instructions. Briefly, for each double-stranded siRNA against one of the four Pnma5 CDS regions, two pairs of synthesized complementary single-stranded DNA oligonucleotides were first annealed to form two double-stranded DNA templates. Subsequently, two complementary single-stranded siRNAs were separately synthesized in accordance with these two templates and then annealed to form a final double-stranded siRNA. Next, the siRNA was purified by conventional phenol/chloroform/isoproponal precipitation, which was then aliquoted and stored at -80°C after a quality check on the agarose gel. A ready-to-use siRNA mixture was prepared by mixing siRNAs against four target regions together at an equal molar ratio to a final concentration of 5 μM.

For siRNA transfection, the N-TER^™^ Nanoparticle siRNA Transfection System (Sigma) was used. Briefly, two tubes, one containing 3 μl N-TERTM nanoparticles in 3.25 μl nuclease-free water (Acros Organics) and the other containing 4.8 μl of siRNA (5 μM) mixture in 1.45 μl of siRNA dilution buffer (provided by the kit) were set up; they were then gently mixed together and incubated at room temperature (RT) for 20 min. Next, the siRNA–nanoparticle complex solution was added into a 100-μl medium drop containing 50 oocytes. After a 12–14 h treatment, the oocytes were washed to remove the nanoparticle-containing medium. After a period of 1–2 h, another one or two rounds of siRNA treatment was performed, depending on how difficult the target was to significantly knock down. During the whole siRNA treatment, typically 36–44 h long, 2.5 nM milrinone was included to prevent resumption of meiosis. Next, oocytes were transferred into milrinone-free MEM+ and cultured for 8 or 16 h. They were then used for the phenotype analysis-related experiment described below.

### In vitro fertilization

Oocytes were transfected and cultured to MII stage as described. Shortly before fertilization, oocytes were washed rapidly for 3 times with MEM+ medium to remove FBS. Spermatozoa were obtained from the epididymis of 10-18 weeks old B6D2F1 male mice and were capacitated (1 h) in 1ml MEM+. Subsequently, 10 μl of a sperm suspension containing 5-10 x 10^6^ spermatozoa/ml was added to 490 ul MEM+ medium, then FBS-free oocytes were added. 9h later, the oocytes were used for immunofluorescence staining to determine normal fertilized or not, by the identification of the two pronuclei.

### Immunofluorescence staining

Oocytes were briefly washed in PBS with 0.05% polyvinylpyrrolidone (PVP), permeated in 0.5% Triton X-100 / PHEM (60 mM PIPES, 25 mM Hepes pH 6.9, 10 mM EGTA, 8 mM MgSO4) for 5 min and washed three times rapidly in PBS / PVP. Next the oocytes were fixed in 3.7% paraformaldehyde (PFA) / PHEM for 20 min, washed three times (10 min each) in PBS / PVP and blocked with blocking buffer (1% BSA / PHEM with 100 mM glycine) at room temperature for 1 h. Then the oocytes were in sequence incubated at 4°C overnight with primary antibody diluted in blocking buffer, washed three times (10 min each) in PBS with 0.05% tween-20 (PBST), incubated at room temperature for 45 min with secondary antibody diluted in blocking buffer (2 μg/ ml in all cases), washed three times (10 min each) in PBST. Finally DNA was stained by 10 μg / ml Hochest 33258 (Sigma) and the oocytes were mounted onto a slide with mounting medium (0.5% propal gallate, 0.1 M Tris-Hcl, PH7.4, 88% Glycerol) and covered with a cover glass (0.13–0.17 μm thick). To maintain the dimension of the oocytes, two strips of double-stick tap (90 μm thick) were sticked between the slide and cover glass. Dilution of primary antibody are as follows: anti-Pnma5, 1:200; anti-p-Pnma5, 1:200; anti-β-tubulin, 1:500; anti acetylated α-tubulin, 1:500, anti-human centromere, 1:500. anti-p-Akt, 1:200, anti-p-Gsk3β, 1:200. The oocytes were examined with an Andor Revolution spining disk confocal workstation (Oxford instruments, Belfast, Northern Ireland). F-actin was stained with 30 nM phalloidin (Cytoskeleton Inc, Denver, USA. Cat no: PHDR1,) together with secondary antibody for 45 min.

### Western blot analysis

A total of 100-200 mouse oocytes were placed in 5^*^SDS sample buffer and heated at 100 °C for 5 min,then cooled on ice and centrifuged at 14000 rpm for 5 min to remove pellets. Proteins were separated by SDS-PAGE with a 4% stacking gel and a 10% separating gel for 1.5 h at 100 voltage (V) and then electrophoretically transferred to polyvinylidene fluoride membranes for 1.5 h at 100 V at 4 °C. After being washed three times (10 min each time) in TBS (20 mM Tris, 137 mM NaCl, pH 7.4),Then membranes were blocked in TBST (TBS with 0.1% Tween 20) containing 5% non-fat milk for 1 h at room temperature, followed by incubation at 4 °C overnight with primary antibody. The next day, after washing three times in TBST (10 min each), membranes were incubated at room temperature for 1 h with horseradish peroxidase-conjugated Pierce anti-mouse IgG (1:5,000) or anti-Rabbit IgG (1:5000), followed by three times washing in TBST(10 min each). Finally signal detection was performed by the enhanced chemiluminescence (ECL) technique using ECL Advance reagents (Amersham Biosciences UK Limited, Little Chalfont Buckinghamshire, England).

### Immunoprecipitation

For immunoprecipitation experiments, 5 μg control IgG or anti-Pnma5 antibody was first coupled to 30 μl protein-A/G beads (Macgene) for 4 h at 4 °C on a rotating wheel in 250 μl IP buffer (20 mM Tris-HCl pH 8.0, 10 mM EDTA, 1 mM EGTA, 150 mM NaCl, 0.05% Triton X-100, 0.05% Nonidet P-40, 1 mM phenylmethylsulfonyl fluoride) with 1:100 protease inhibitor (Sigma) and 1:500 phosphatase inhibitor (Sigma). Meanwhile, 600 oocytes cultured for 8 h *in vitro* to MI Stage were lysed and ultra-sonicated in 250 IP buffer and then pre-cleaned with 30 ul protein-A/G beads for 4 h at 4 °C. Then, protein A/G-coupled control IgG or anti-Pnma5 antibody was incubated overnight at 4 °C with 250 μl pre-cleaned oocyte lysate supernatant. Finally, the next day beads were washed three times for 10 min each with 1 ml IP buffer and the resulting beads with bound immuno complexes were subjected to SDS-PAGE and silver staining.

### Chromosome spread

Oocytes were exposed to Tyrode’s buffer (pH 2.5) for 40-50 secondes to remove zona pellucidae, and then fixed in a drop of 1% paraformal -dehyde with 0.15% Triton X-100 on a glass slide. Kinetochores and chromosomes were then stained as that of immunofluorescence. Andor Revolution spining disk confocal workstation was used to examine chromosome and kinetochore numbers in oocytes.

### Mitochondrial staining and ATP measurements

For mitochondrial staining, the oocytes were stained in Hepes containing 100 nM Mito Tracker (Invitrogen, m7521) for 30 minutes. For measurement of ATP, the oocytes were first lysed with 100 μl RIPA lysis solution on ice. The samples were then detected by enzyme-labeled instrument Synergy2 (BioTek, USA) to evaluate ATP level.

### Silver staining and characterization of Pnma5-interacting proteins

For silver staining, immuno-complexed beads from control IgG or anti-Pnma5 antibody group were boiled in protein sample buffer and the supernatants were separated side by side on a SDS-PAGE gel and the gel were subsequently fixed overnight at 4°C in fixing solution (10% acetic acid, 40% ethanol), sensitized 30 min at room temperature with freshly-prepared sensitizing solution (30% ethanol, 0.2% Na_2_S_2_O_3_, 0.314% Na_2_S_2_O_3·_5H_2_O and 6.8% sodium acetate) and washed three times with water for 5 min each. Then the gel was stained for 20 min at room temperature in fresh-made staining solution (0.25% AgNO_3_, 0.02% of fresh 37% formaldehyde solution), washed with water for 2.5 min and developed for about 5-10 min (depending on how fast the process is, avoid insufficient or excessive development) in developing solution (2.5% NaCO_3_, 0.02% of fresh 37% formaldehyde solution) and finally the developing reaction was stopped for 10 min in stopping solution (0.4% glycine).

To identify Pnma5-interacting proteins, silver-stained control or Pnma5 lanes were compared carefully and those bands with significantly higher gray level in Pnma5 lane were cut out one by one and stored in protease-free tubes with 10% ethanol. Then the selected bands, which were potentially Pnma5 interactors, were sent to Testing & Analysis Center, Nanjing Medical University for MALDI-TOF-MS (Matrix-Assisted Laser Desorption / Ionization Time of Flight Mass Spectrometry). The identity of each protein was determined by PMF (peptide mass fingerprinting) searching in Mascot (http://www.matrixscience.com/mascot/cgi/search_form.pl?FORMVER=2&SEARCH=PMF).

### Identification of pnma5 phosphorylation site and generation of antibody specific to p-Pnma5

Generally, the phosphorylated part of a protein is about 0.01- 0.1%, so it’s almost impossible to use oocytes for identification of phosphorylation sites. Therefore, we used NIH3T3 cells instead, then generated phospho-specific antibody and verify the antibody in oocytes. We used 5 IP reactions, each of which employed 1x10^6^ NIH3T3 cells, 30 μl protein A/G beads and 5 μg Pnma5 antibody. Then the immuno-complexed beads were eluted by 0.2 M glycine (pH 2.7), and the phosphorylated portion of the immuno-complex was enriched by Pierce™ TiO_2_ Phosphopeptide Enrichment and Clean-up Kit (Thermo Scientific, Rockford, IL) and sent to Testing & Analysis Center, Nanjing Medical University for LC-MS (liquid chromatograph-mass spectrometer). We pick Thr553 (phosphorylation possibility 93.4%) and the whole process of antibody production & purification was performed by Zhong Ding Biotechnique, Ltd (Nanjing, Jiangsu, China). A short phospho peptide MPTGT(ph)EAAQGVE (“C” at the C-term is an extra residue for conjugation) was synthesized and injected into rabbit for the serum production. The phospho-specific antibody was purified from the serum through column filled with phospho peptide-conjugated resin and then absorbed through column filled with non-phospho peptide (MPTGTEAAQGVE)-conjugated resin to remove residual non-phospho-specific antibody.

### Data analysis and statistics

All experiments were repeated at least three times, Measurement on confocal Images was done with Image J. Data were presented as average ± Sem. Statistical comparisons between two groups were made with Student’s test of EXCEL, statistical comparisons between multiple groups were made with a one-way nonparametric analysis of variance (Kruskal-Wallis) (GraphPad Prism; GraphPad Software). P<0.05 was considered to be statistically significant.

## Abbreviations

IVM: *in vitro* maturation
IVF: *in vitro* fertilization
LH: luteinizing hormone
PNMA: paraneoplastic Ma
PDAC: pancreatic ductal adenocarcinoma
PND: post-natal day
GCs: granulosa cells
GV: germinal vesicle
PN: pronucleus
IP: immunoprecipitate
MALDI-TOF-MS: Matrix-Assisted Laser Desorption/ Ionization Time of Flight Mass Spectrometryl
LC-MS: liquid chromatograph-mass spectrometer
